# Green, Formaldehyde-Free Bio-Adhesive from Soybean Meal and Laccase-Oxidized Tannin via Quinone–Amine Crosslinking

**DOI:** 10.3390/polym18080954

**Published:** 2026-04-14

**Authors:** Shichao Zhang, Chengyuan Liu, Ya Ding, Yuan Yao, Hisham Essway, Xinyi Chen, Xiaojian Zhou, Hui Wang, Ming Cao

**Affiliations:** 1 Yunnan Provincial Key Laboratory of Wood and Bamboo Biomass Materials, Southwest Forestry University, Kunming 650224, China; 2 International Joint Research Center for Biomass Material, Southwest Forestry University, Ministry of Science and Technology, Kunming 650224, China; 3 Department of Polymers and Pigments, National Research Centre, Cairo 12622, Egypt

**Keywords:** bio-based adhesive, laccase-oxidized tannin, soybean meal, quinone–amine cross-linked network, thermal stability, water resistance

## Abstract

To develop a fully green and non-toxic wood adhesive with improved water resistance and bonding performance for soybean meal (*Glycine max* (L.) Merr.)-based adhesives, oxidized tannin (OTN) was obtained by the laccase treatment of waxberry tannin (TN), a natural polyphenolic polymer, and then blended with soybean meal (SM) to prepare an oxidized tannin–soybean meal adhesive (OTS). Laccase-mediated oxidation converted the tannin polymer into quinone-rich oxidized polymeric structures, which reacted with amino groups in soybean meal proteins through Michael addition and Schiff base reactions to form a covalently crosslinked polymeric network. Under the optimal conditions of a laccase dosage of 10%, an oxidation time of 6 h, an OTN:SM mass ratio of 0.5:1, and a hot-pressing temperature of 160 °C, plywood bonded with OTS exhibited a wet shear strength of 0.85 MPa at 63 °C, representing a 136% increase over that of the neat soybean meal adhesive, and showed slightly higher bonding performance than the commercial urea-formaldehyde (UF) resin under boiling-water conditions. Structural analyses (FT-IR and XPS) verified quinone formation and carbon–nitrogen single and double bonds. Thermal analyses (DSC and TGA) revealed improved curing reactivity and significantly enhanced thermal stability compared with the neat soybean meal adhesive.

## 1. Introduction

Adhesives relying on formaldehyde as a base remain dominant within the wood processing sector. However, as they rely on non-renewable resources, in addition to the harmful effect of the formaldehyde component, they pose threats to the environment. Consequently, considerable research efforts have been directed towards developing sustainable and eco-friendly alternatives derived from biomass materials, such as those based on starch, lignin, proteins, and tannins. Within the spectrum of bio-based options, soy protein adhesives have attracted considerable interest as highly promising candidate. They are derived from renewable and abundant resources, are inherently non-toxic, and exhibit advantages such as low cost and facile processing. However, the inherent molecular structure and strong intermolecular interactions of soy protein often result in wood adhesives with low solid content and high viscosity [1]. Moreover, the hydrophilicity of these groups often results in poor water resistance in the resultant adhesive, thereby constraining its wider adoption and utilization in commercial settings [2,3].

To address these limitations, several modification strategies have been explored. Some relevant studies have focused on optimizing the processing parameters. For instance, Luo et al. [4] found that prolonging the hot-pressing time could promote more thorough curing of soybean meal-based adhesives, thereby enhancing water resistance. A particularly prominent chemical approach involved blending soy protein with tannins, which are rich in phenolic hydroxyl groups, to form cross-linked networks. Ghahri et al. [5] employed a composite tannin system with hexamethylenetetramine as a cross-linking accelerator, and reported enhanced water resistance through covalent interactions. Liu et al. [6] also reported that formulating adhesives from tannins and soy protein under different pH conditions yielded cross-linked structures with improved water resistance and thermal stability. To achieve even greater performance, prior chemical modification of tannins has been pursued. A notable example is the work of Chen et al. [7], who synthesized a phenolic hydroxymethylated prepolymer that formed a dense three-dimensional network with soy protein, drastically improving the wet bond strength. Despite these advancements, significant challenges persist in the application of tannins. Firstly, their high reactivity often leads to intermolecular self-polymerization, consuming active sites, while their bulky, three-dimensional structures create steric hindrance that limits effective contact with protein functional groups [8,9,10]. More critically, many effective modification strategies, including the hydroxymethylation method used by Chen et al. [7], inherently depend on toxic reagents like formaldehyde [11,12]. This reintroduces the environmental and health hazards that bio-based adhesives are meant to avoid. Therefore, developing a green and efficient pathway to activate tannins without toxic chemicals is crucial for the technological upgrading of tannin-modified soy adhesives [13,14].

Enzymatic catalysis using laccase offers a promising solution to this dilemma. Laccase is an environmentally friendly redox enzyme that works under mild conditions, utilizing molecular oxygen as the terminal electron acceptor and reducing it to water as the sole byproduct [15,16]. Its multicopper active center efficiently catalyzes the oxidation of phenolic hydroxyl groups in tannins, converting them into highly reactive quinone structures [17,18,19]. These quinone intermediates can then undergo non-formaldehyde cross-linking reactions, specifically Michael addition and Schiff base formation, with amino groups in soy protein, forming stable carbon–nitrogen covalent bonds [20], thereby creating a sturdy spatial network that boosts the mechanical properties and water resistance. Previous studies have shown that laccase has been applied in several bio-based adhesive systems. For example, Ibrahim et al. used laccase-modified kraft lignin to formulate adhesives with soy protein, chitosan, and polyethyleneimine, demonstrating the feasibility of laccase-mediated lignin activation in adhesive preparation [21]. Pradyawong et al. further reported that laccase/TEMPO-modified lignin improved the adhesion performance and water resistance of soy-protein-based adhesives [22]. In addition, Jimenez Bartolome et al. used enzymatically polymerized lignosulfonates to enhance the wet resistance of starch-based adhesives [23]. Moreover, laccase has also been shown to promote covalent coupling between soy protein and polyphenols. Wang et al. reported that laccase-catalyzed oxidation of gallic acid generated quinone intermediates, which subsequently reacted with soy protein isolate to form covalent complexes through C=N and C–S linkages [24]. These studies provide important evidence for the feasibility of laccase-mediated activation of phenolic substrates and protein–polyphenol coupling in bio-adhesive systems. However, previous laccase-assisted bio-adhesive studies have mainly focused on lignin derivatives, lignosulfonates, or small-molecule phenolics, whereas the use of a natural polymeric tannin as a reactive precursor for soybean meal-based plywood adhesives remains much less explored. In contrast, the present work employs waxberry tannin as a naturally derived polymeric polyphenol and activates it under mild enzymatic conditions without aldehyde-based crosslinkers or additional chemical oxidants. More importantly, the novelty of this study does not merely lie in developing a formaldehyde-free adhesive, but in establishing a waxberry tannin–soybean meal adhesive system based on a specific quinone–amine coupling mechanism. Specifically, laccase-catalyzed oxidation converts waxberry tannin into quinone-rich polymeric structures, which subsequently react with amino groups in soybean meal proteins through Michael addition and Schiff base reactions to form a dense covalently crosslinked network.

Based on the above considerations, this study developed a formaldehyde-free enzymatic route to oxidize waxberry tannin, a natural tannin polymer, with laccase and construct a soybean meal-based adhesive system. A dense covalently crosslinked network was proposed to form through quinone–amine reactions, including Michael addition and Schiff base reactions, between oxidized tannin and amino groups in soybean meal proteins. To verify this hypothesis, the effects of laccase dosage, oxidation time, OTN/SM mass ratio, and hot-pressing temperature on the shear strength and water resistance of plywood were systematically investigated. In addition to wet shear strength, the hydrolysis residual ratio was introduced as a supplementary index for overall performance evaluation, and the optimal preparation conditions were established accordingly. Furthermore, FT-IR and XPS analyses were performed to elucidate the reaction mechanism, while DSC and TG were used to characterize the curing behavior and thermal properties of the adhesive.

## 2. Materials and Methods

### 2.1. Materials

Commercial veneer from poplar (*Populus tomentosa* Carr.), with its water content maintained at 8–10%, was employed in the experiments. Waxberry tannin (*Myrica rubra* (Lour.) Siebold & Zucc.) (industrial grade, tannin content 72%, average molecular weight of approximately 2292 Da), soybean meal powder, and citric acid–sodium citrate buffer were sourced from the Wuming Tannin Extract Factory (Nanning, China), Shandong Yuxin Biotechnology Co., Ltd. (Binzhou, China), and Guangdong Guanghua Sci-Tech Co., Ltd. (Shantou, China), respectively. Laccase (100 U/mL, food grade, derived from white–rot fungi) was purchased from Angsheng Chemical Co., Ltd. (Yaan, China). A commercial urea-formaldehyde (UF) resin, with a solid content of 55% and an F/U molar ratio of 1.2, was supplied by Langfang Senbang Chemical Co., Ltd. (Langfang, China).

### 2.2. Preparation of OTN and OTS

For the preparation of a 40% (*w*/*v*) TN solution, predetermined amounts of tannin and citric acid–sodium citrate buffer (pH 4.5) were sequentially transferred to a three-neck flask fitted with a mechanical stirrer and a condenser. Subsequently, laccase equivalent to 10% of the mass of solid TN was incorporated into the solution. The solution was subjected to mechanical stirring and subsequently heated using a thermostatically controlled water bath set to 40 °C over a predetermined duration. Following the oxidation reaction, the mixture was chilled to 23 ± 3 °C to obtain the oxidized tannin (OTN) solution. The final solution was cooled and freeze-dried to produce the oxidized tannin (OTN) sample.

**Figure 1:**
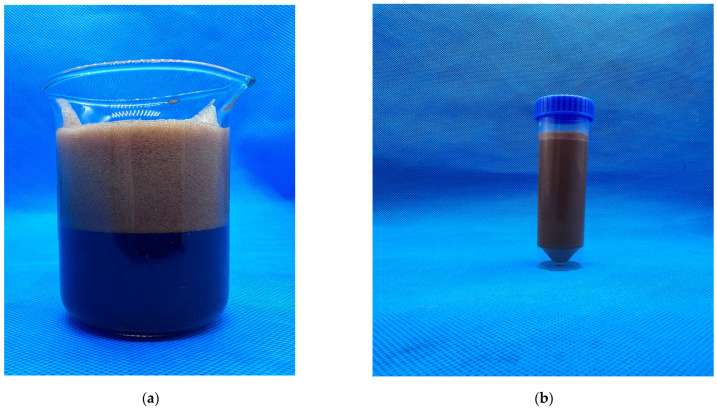
Photographs of the appearance of OTN and OTS: (**a**) OTN solution; (**b**) OTS.

At room temperature, the OTS was formulated by mixing OTN, SM, and deionized water, with the mass ratio of the solid components (OTN + SM) to deionized water set at 27.93:100. The blend was mechanically stirred at 400 rpm for 30 min to ensure the homogeneous mixing of the solids. The resulting OTS adhesive had a solid content of 21%. In addition to investigating the effects of laccase addition, oxidation time, the mass ratio of OTN to SM, and hot-pressing conditions on the bonding performance of the adhesive using a single-variable approach, a preliminary evaluation of several industrially relevant parameters was also carried out for the freshly prepared OTS adhesive, including viscosity, coating behavior, and pot life at different times after preparation. As shown in Table 1, the adhesive exhibited an initial viscosity of 303 mPa·s and could be easily applied with a uniform coating immediately after preparation; after 1.0 h, the viscosity increased to 596 mPa·s and the coating behavior became less uniform; after 1.5 h, caking occurred and the adhesive could no longer be applied (Figure 1).

### 2.3. Structural Analysis

The curing of the OTS was carried out at 160 °C for a duration of 3 h. The cured OTS adhesive, along with TN, SM, and freeze-dried OTN, were individually ground into powders for Fourier transform infrared spectroscopy (FT-IR) analysis. Each sample was intimately ground with KBr in a 1:100 ratio by mass, then compressed to form pellets. Spectral data were recorded using a Thermo Nicolet 670 instrument (Thermo Fisher Scientific, Waltham, MA, USA) at a resolution of 4 cm^−1^ with 32 scan accumulations, covering the wavenumber region from 4000 to 500 cm^−1^.

The samples, including TN, SM, freeze-dried OTN, and cured OTS, were pulverized for X-ray photoelectron spectroscopy (XPS) analysis. A Thermo Scientific K-Alpha spectrometer (Thermo Fisher Scientific, Waltham, MA, USA), equipped with a monochromatic Al Kα X-ray source (hv = 1486.6 eV), was employed for XPS analysis. Data fitting and analysis were performed using Advantage 6.10.0 software, with calibrations aligned with the adventitious carbon C1s signal set to 284.8 eV.

### 2.4. Thermal Analysis

Differential scanning calorimetry (DSC) was conducted on the OTS using a Netzsch 204 F1 instrument (Netzsch, Selb, Germany) under a stream of nitrogen maintained at 21 mL/min. Samples (4–5 mg) inside hermetic aluminum pans were scanned from 30 to 250 °C using a ramp rate of 10 °C/min to monitor its curing behavior.

Thermogravimetric analysis (TGA) of the OTS was carried out using a Netzsch TG 209 F1 instrument (Netzsch, Selb, Germany). Liquid adhesive samples (5–6 mg) were heated from 35 to 600 °C at a constant rate of 10 °C/min under a nitrogen atmosphere (50 mL/min).

### 2.5. Adhesive Water Stability

The water stability of the cured adhesive was characterized by the hydrolysis residual ratio. This index reflects the proportion of adhesive that remains after hydrolysis in hot water and can therefore be used to evaluate the resistance of the cured adhesive network to hydrolytic degradation [20,25]. Generally, a higher hydrolysis residual ratio corresponds to better water stability of the adhesive.

Following curing at 160 ± 3 °C, the adhesive was pulverized to pass through a 200-mesh sieve. A precisely weighed portion (m_1_) was enclosed in an ashless filter paper and subjected to hydrolysis in water at 63 ± 3 °C lasting 3 h. Subsequent to drying at 80 ± 3 °C to achieve constant mass (m_2_), its hydrolysis residual ratio was calculated.

$$\mathrm{Hydrolysis}\;\mathrm{residual}\;\mathrm{ratio}\;(\%)=\frac{m_{2}}{m_{1}}\;\times \;100\%$$

### 2.6. Plywood Preparation and Adhesive Mechanical Properties

Poplar veneer, measuring 200 mm in length, 130 mm in width, and 2 mm in thickness, was used to fabricate three-layer plywood. After thorough stirring, only the OTS was uniformly applied by manual coating to both surfaces of the core veneer, with a spread rate of 300 g/m^2^ on each surface. The surface veneers were arranged perpendicular to the grain direction of the core veneer and pre-pressed at 1.0 MPa for 30 min under ambient conditions. Subsequently, the assembled veneers were hot-pressed in a single-platen press at 160 °C and 1.2 MPa for 7 min. After hot pressing, the plywood was conditioned at 23 °C for 24 h to release residual stresses prior to mechanical testing.

**Scheme 1:**
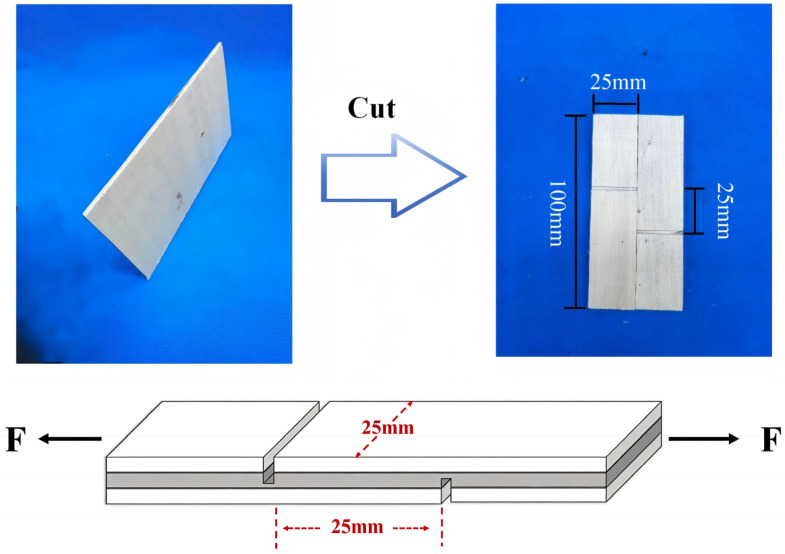
Preparation, dimensions, and loading direction of the bonded wood specimens for shear strength testing.

Shear specimens measuring 100 mm by 25 mm were prepared as illustrated in Scheme 1. The shear strength was evaluated under four conditions, namely dry, after 24 h immersion in cold water (20 ± 3 °C), after 3 h immersion in hot water (63 ± 3 °C), and after 3 h immersion in boiling water, following the method outlined in GB/T 17657-2022 [26]. The maximum load at failure was determined using a WDS-50KN universal testing machine. Each measurement was performed with nine independent replicates, and the shear strength was calculated as the mean value.

## 3. Results

### 3.1. Structural Analysis of TN and OTN

**Figure 2:**
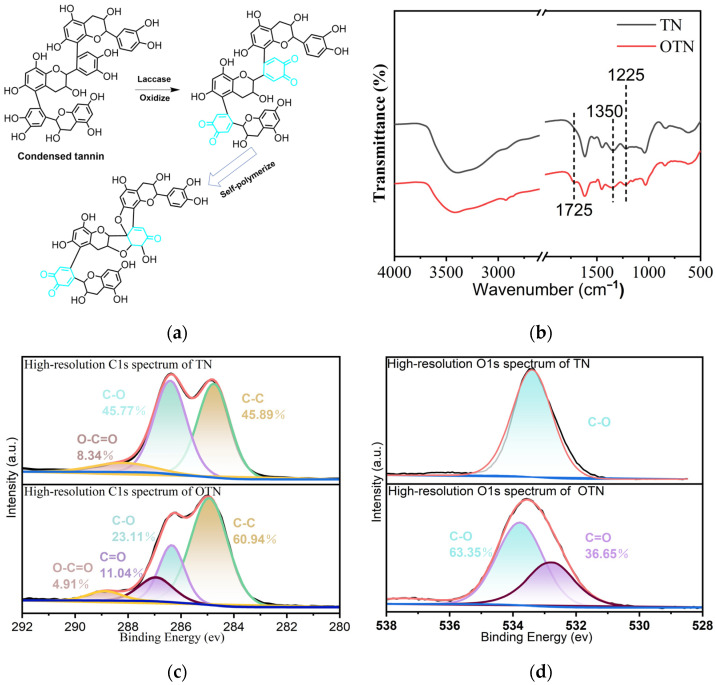
Structural characterization of TN and OTN: (**a**) schematic illustration of the possible laccase-catalyzed oxidation pathway of tannin; (**b**) FT-IR spectra of TN and OTN; (**c**) high-resolution C1s XPS spectra of TN and OTN; (**d**) high-resolution O1s XPS spectra of TN and OTN.

The hypothetical mechanism diagram of laccase-catalyzed oxidation of tannin is shown in Figure 2a. The investigation accomplished using FT-IR and XPS collectively confirmed the successful introduction of quinone structures into the tannins via laccase-catalyzed oxidation. As observed in the FT-IR spectrum for oxidized tannin (OTN) (Figure 2b), a novel peak emerges at 1725 cm^−1^. This signal is assigned to the C=O stretching vibration of carbonyl groups present in this quinone structure. Concurrently, a broadening noted in the C–O alongside aromatic C–O–C vibrational peaks at 1350 cm^−1^ and 1225 cm^−1^ further indicated the oxidation of phenolic hydroxyl groups and their potential involvement in forming quinone structures or new ether/ester linkages [27]. These observations were additionally corroborated by XPS results. In Figure 2c, the C1s spectrum of OTN displayed a distinct peak at 287.02 eV (11.04%), characteristic of quinone carbonyl (C=O), while in Figure 2d, the corresponding O1s spectrum showed a peak at 532.68 eV (36.65%), assigned to quinone oxygen, thereby unequivocally verifying quinone formation [28]. Nevertheless, a significant C–O signal (23.11%) persisted at 286.21 eV in the C1s spectrum, along with a prominent hydroxyl-related peak at 533.78 eV (63.35%) in the O1s spectrum, indicating that a substantial proportion of phenolic hydroxyl groups remained unoxidized. This incomplete conversion is likely attributable to steric hindrance effects imposed by the three-dimensional configuration of tannin molecules, which limits the accessibility of internal phenolic hydroxyl groups to laccase, thereby constraining oxidation efficiency [29]. Collectively, the FT-IR and XPS results support that laccase oxidation introduced quinone structures into the tannin polymer, and that these oxidized polymeric structures further reacted with soybean meal during curing.

### 3.2. Structural Analysis of the OTSs

**Figure 3:**
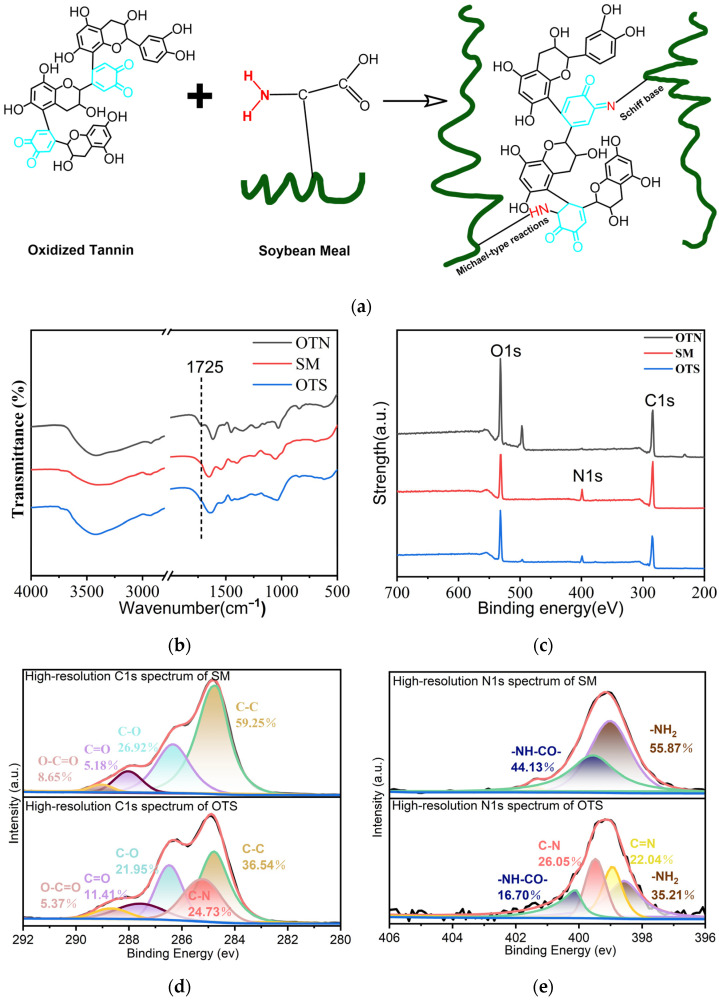
Structural characterization of OTS: (**a**) schematic illustration of the proposed quinone–amine crosslinking mechanism of the OTS adhesive; (**b**) FT-IR spectra of OTN, SM, and the OTS adhesive; (**c**) XPS full spectra of OTN, SM, and the OTS adhesive; (**d**) high-resolution C1s XPS spectra of SM and the OTS adhesive; (**e**) high-resolution N1s XPS spectra of SM and the OTS adhesive.

The hypothesized crosslinking mechanism of OTS is shown in Figure 3a. To further elucidate the reaction mechanism of the oxidized tannin–soybean meal adhesive (OTS), systematic analysis of the chemical composition for OTN, SM, and OTS was conducted via FT-IR and XPS. According to the FT-IR spectra presented in Figure 3b, the peak intensity of the quinone carbonyl spectral feature at 1725 cm^−1^ was markedly reduced in the OTS sample, indicating most of the quinone groups were consumed during cross-linking [30]. The chemical composition of OTN, the OTS adhesive, and SM was analyzed using XPS. The full-range XPS spectra corresponding to these materials are depicted in Figure 3c, while the resulting elemental compositions are summarized in Table 2. The full-spectrum confirmed that carbon, oxygen, and nitrogen are the primary constituents of the three materials. The nitrogen content in case of OTS (5.69%) falls between that of OTN (3.02%) and SM (7.68%), which is consistent with the successful incorporation of SM into the adhesive and its participation in the cross-linking reaction. The trace nitrogen detected in OTN likely originated from impurities. Further, the high-resolution spectra provided direct evidence of covalent cross-linking. The C1s spectrum of OTS (Figure 3d) revealed a newly emerged peak positioned at 285.28 eV, assigned to C-N bonds, indicative of quinone–amine interactions. More notably, the N1s spectrum (Figure 3e) was accompanied by the emergence of two new peaks at 398.88 eV and 399.48 eV, indicative of C=N (Schiff base) and C-N (Michael addition) bonds, respectively. These results confirm the consumption of amino groups via both covalent pathways. However, the residual peak corresponding to free amino groups at 399.58 eV indicated an incomplete reaction. Collectively, the FT-IR and XPS investigations demonstrate that the laccase-catalyzed pathway involved oxidation of phenolic hydroxyl groups to quinones [31], which subsequently underwent reaction with the amino functionalities within soy protein through Michael addition and Schiff base reactions in the OTS adhesive, yielding a robust cross-linked network structure. These findings indicate that laccase-oxidized tannin can covalently react with soybean meal through quinone–amine chemistry during curing. The concurrent detection of consumed quinone carbonyls (FT-IR) and the generation of newly formed C-N and C=N bonds (XPS) substantiates the proposed reaction mechanism.

### 3.3. Adhesive Mechanical Properties

The performance of OTS was characterized with respect to shear strength along with the residual ratio of plywood formulated with the OTS adhesives.

#### 3.3.1. Laccase Dosage

**Figure 4:**
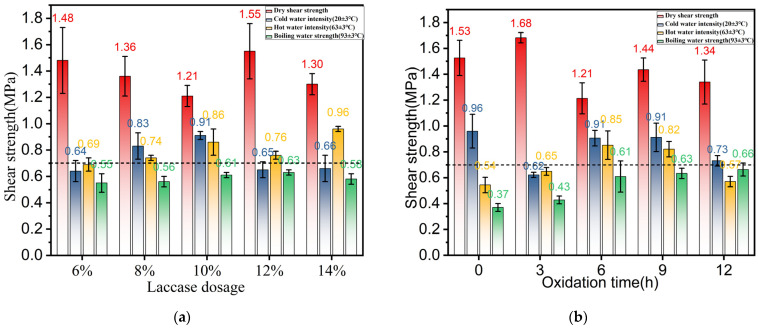

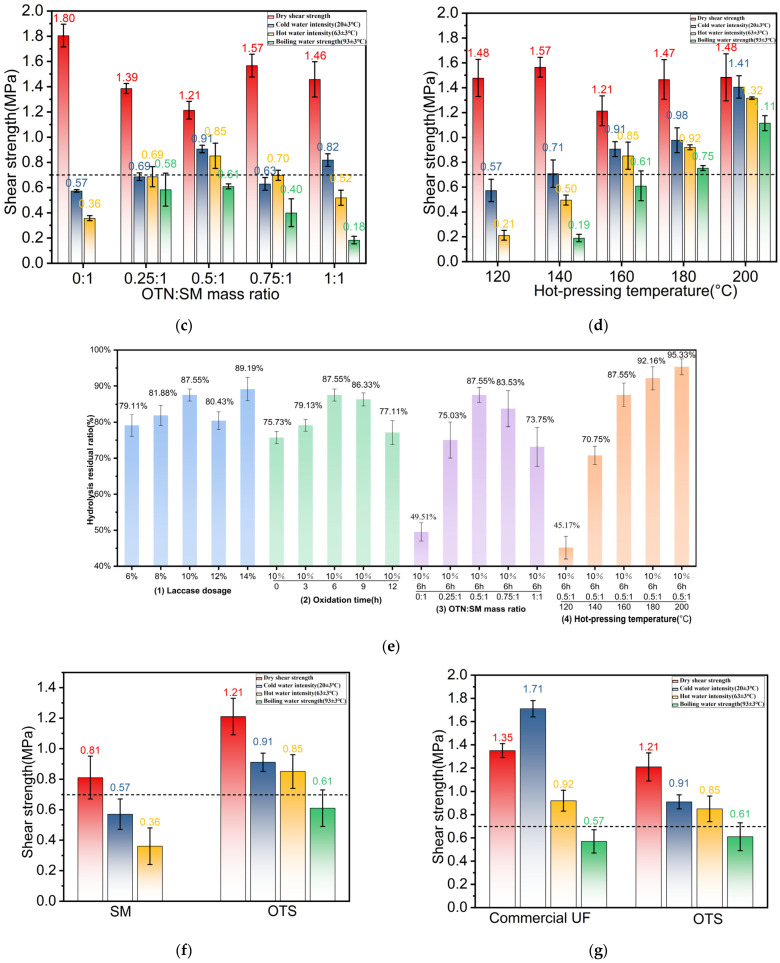
Performance evaluation of OTS: (**a**) effect of laccase dosage on the shear strength of plywood bonded with the OTS adhesive; (**b**) effect of oxidation time on the shear strength of plywood bonded with the OTS adhesive; (**c**) effect of the OTN:SM mass ratio on the shear strength of plywood bonded with the OTS adhesive; (**d**) effect of hot-pressing temperature on the shear strength of plywood bonded with the OTS adhesive; (**e**) effects of laccase dosage, oxidation time, OTN:SM mass ratio, and hot-pressing temperature on the hydrolysis residual ratio of the OTS adhesive; (**f**) comparison of the shear strength of plywood bonded with SM and OTS; (**g**) comparison of the shear strength of plywood bonded with commercial UF and OTS adhesives.

How laccase dosage affects the adhesive performance of the OTS can be seen in Figure 4a. The experiments were carried out using preliminary experimental conditions: oxidation temperature, 40 °C; oxidation time, 6 h; OTN:SM mass ratio, 0.5:1; hot-pressing temperature, 160 °C. The dry shear strength peaked at 1.55 MPa with 12% laccase dosage, however, decreased to 1.30 MPa at 14% dosage. This suggests that excessive laccase may induce over-oxidation and self-polymerization of tannin molecules [32]. The self-polymerization process consumes active quinone groups that would otherwise react alongside amino groups within soy protein, thereby affecting quinone–amine network integrity.

After 24 h cold-water immersion, the bonding performance showed a similar trend, with the shear strength reaching a maximum of 0.91 MPa at 10% laccase dosage, then dropping to 0.65 MPa at a 12% dosage and to 0.66 MPa at a 14% dosage, respectively. The optimal performance acquired at 10% laccase indicates a well-balanced crosslinking density at this level.

After 3 h immersion in water at 63 °C, the bonding strength displayed a complicated trend as it increased to 0.86 MPa at 10% dosage, then decreased, before unexpectedly recovering to 0.96 MPa at 14% dosage. Notably, the hydrolysis residual ratio shown in Figure 4e(1) followed a generally consistent trend with the hot-water shear strength, both peaking at 14% laccase dosage. The highest residual ratio corresponds well with the highest hot-water shear strength observed here. At high laccase dosage, a self-polymerized network forms among tannin molecules [32], which provides sufficient short-term resistance to warm-water hydrolysis. However, this self-polymerized structure exhibits insufficient long-term water resistance, as corroborated by the decrease in shear strength after 24 h cold-water immersion.

Considering the overall performance and stability, the formulation with 10% laccase dosage demonstrated high shear strength and excellent water resistance under both cold- and hot-water conditions, while maintaining a relatively high hydrolysis residual ratio. Therefore, 10% laccase was selected as the optimal dosage for subsequent formulation.

#### 3.3.2. Laccase Oxidation Time

Under fixed conditions of oxidation temperature, 40 °C; laccase dosage, 10%; OTN:SM mass ratio, 0.5:1; and hot-pressing temperature, 160 °C, the oxidation time of tannin was identified as a critical factor affecting the bonding properties. As illustrated in Figure 4b, relative to the adhesive prepared from unoxidized tannin, the bonding strength of lap-shear plywood specimens following a 3 h soak in 63 °C hot water first increased and then decreased with prolonged oxidation time. The best bonding performance in hot water, corresponding to a shear strength of 0.85 MPa, was obtained after 6 h of oxidation. This is attributed to the high content of quinone groups formed during oxidation, which enhanced the reactivity of the system. Under hydrothermal conditions, these quinones react with amino groups to form a more compact crosslinked network, thereby improving the water resistance.

In contrast, the shear strength after 3 h immersion in boiling water exhibited a continuous increasing trend with increasing oxidation time. At an oxidation time of 12 h, strength increased to 0.66 MPa, representing a 53.50% improvement compared with the adhesive prepared with unoxidized tannin. This phenomenon indicates that during prolonged oxidation (e.g., 9 and 12 h), significant self-polymerization occurs among the tannin molecules, forming an internal network connected via C–C or C–O–C bonds [33]. However, the interaction between this self-polymerized network and the soy protein matrix primarily relies on a weaker hydrogen bonds, physical interactions or mechanical interlocking, rather than stable quinone–amine covalent cross-links. From the perspective of the entire adhesive system, this self-polymerized tannin essentially is incorporated into the soy protein matrix mainly through physical entanglement and non-covalent interactions. Such a rigid network can effectively resist the penetration and mechanical stress of boiling water in the short term, thus exhibiting higher immediate strength under extreme testing conditions. Nevertheless, due to the lack of toughness and structural stability imparted by quinone–amine covalent bonds, the long-term durability of this self-polymerization-dominated network is poor. This inference is consistent with the observations in Figure 4e(2), which reveals that beyond 6 h of oxidation, adhesive hydrolysis residual ratio gradually declined, further confirming the insufficient stability of its crosslinked network under long-term hydrothermal conditions. Therefore, 6 h was selected as the optimal oxidation time for OTN preparation.

#### 3.3.3. OTN:SM Mass Ratio

The mass ratio between OTN and SM constitutes a key parameter that critically influences both the structure and the resulting performance of OTS. Presented in Figure 4c are the shear strength outcomes of plywood specimens fabricated using different OTN:SM mass ratios while other conditions were kept unchanged: oxidation temperature, 40 °C; laccase dosage, 10%; oxidation time, 6 h; and hot-pressing temperature, 160 °C. The corresponding hydrolysis residual ratios are presented in Figure 4e(3) as well. It is implied that when no tannin was added, the adhesive exhibited low wet shear strength, while at an OTN:SM mass ratio of 0.5:1, the cold-water shear strength of 0.91 MPa was achieved, representing a 59.30% improvement over the pure SM adhesive. Meanwhile, the hot-water (63 °C) shear strength peaked at approximately 0.85 MPa, accounting for a 136% increase, and the boiling-water shear strength reached 0.61 MPa, accompanied by a hydrolysis residual ratio of 87.55%. These results indicate the formation of an efficient quinone–amine cross-linked network between OTN and SM at this mass ratio. On the one hand, sufficient quinone groups were available to react with amino groups in the protein, which significantly enhanced the crosslinking density. Conversely, the soy meal protein matrix provides adequate support for these crosslinking points, producing a composite that is both strong and tough.

When this OTN:SM mass ratio exceeded 0.75:1, the shear strength and hydrolysis residual ratio of the plywood gradually declined, which can be correlated with the fact that tannin molecules inherently contain a large number of phenolic hydroxyl groups. Even after partial oxidation, the presence of excessive content of residual hydrophilic groups increases the overall hydrophilicity of the system, thereby impairing water resistance to some extent. Therefore, optimum performance was established for an OTN:SM mass ratio of 0.5:1.

#### 3.3.4. Hot-Pressing Temperature

This study examined how hot-pressing temperature affected the shear strength of plywood, while keeping the other conditions fixed: oxidation temperature, 40 °C; laccase dosage, 10%; oxidation time, 6 h; and OTN:SM mass ratio, 0.5:1. The results are shown in Figure 4d.

A general increasing trend regarding wet shear strength was evident with increasing hot-pressing temperature. At 120 °C, the shear strength values for plywood attained values of 1.48 MPa (dry), 0.57 MPa (cold water), and 0.21 MPa (hot water). After immersion in boiling water, the plywood failed to maintain structural integrity, indicating insufficient cross-link along with incomplete curing process for the adhesive under this temperature condition. At a hot-pressing temperature of 140 °C, the adhesive began to exhibit measurable boiling-water shear strength, suggesting promotion in crosslinking reactions and improved network formation. At 160 °C, both the cold- and hot-water shear strengths complied with the Type II requirements set forth by the Chinese National Standard GB/T 17657-2022 [26]. A further temperature increase to 200 °C led to a significant enhancement in shear strength: 1.41 MPa (cold water), 1.32 MPa (hot water), and 1.11 MPa (boiling water). These results indicate that increasing the hot-pressing temperature promotes quinone–amine cross-linking and leads to a more compact cured network.

Meanwhile, the hydrolysis residual ratios of the OTS at different hot-pressing temperatures are shown in Figure 4e(4), which revealed a continuous increase in the hydrolysis residual ratio with the temperature, reaching 95.33% at 200 °C. This indicates that a more compact crosslinked structure was formed in the cured system at higher hot-pressing temperatures, effectively inhibiting the erosion caused by the penetration of water molecules, thereby exhibiting excellent hydrolysis resistance.

Based on the overall bonding performance and curing efficiency, 160 °C was selected as the optimal hot-pressing temperature for plywood preparation.

For comparison, plywood specimens bonded with neat soybean meal (SM) adhesive and commercial UF adhesive were also prepared using the same glue spread and hot-pressing conditions as those used for the OTS. Figure 4f,g show the shear strengths of plywood bonded with commercial UF, neat soybean meal (SM), and OTS under different testing conditions. Under dry conditions, the plywood bonded with the OTS adhesive exhibited a shear strength of 1.21 MPa, which was slightly lower than that of the commercial UF adhesive (1.35 MPa) but significantly higher than that of the neat SM adhesive (0.81 MPa), corresponding to an increase of 49.4%. After 24 h cold-water immersion, the OTS-bonded plywood retained a shear strength of 0.91 MPa, which was lower than that of the commercial UF adhesive (1.71 MPa) but still much higher than that of the neat SM adhesive (0.57 MPa), representing an improvement of 59.6%. Under the more severe hot-water condition at 63 °C, the OTS adhesive still maintained a shear strength of 0.85 MPa, which was markedly higher than that of the neat SM adhesive (0.36 MPa) and close to that of the commercial UF adhesive (0.92 MPa), corresponding to a 136.1% increase over the neat SM adhesive. After boiling-water treatment, the OTS adhesive retained a shear strength of 0.61 MPa, which was slightly higher than that of the commercial UF adhesive (0.57 MPa), whereas the plywood bonded with the neat SM adhesive failed after boiling-water treatment and no measurable bonding strength was obtained. Overall, these results demonstrate that the OTS adhesive provides markedly improved bonding strength and wet durability compared with the neat SM adhesive, while achieving bonding performance comparable to that of the commercial UF adhesive under harsh hydrothermal conditions.

### 3.4. Thermal Analysis of SM and OTS

**Figure 5:**
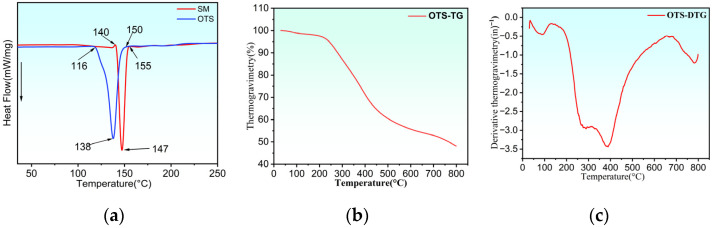
Thermal characterization of OTS: (**a**) DSC curves of SM and the OTS adhesive; (**b**) TG curve of the OTS adhesive; (**c**) DTG curve of the OTS adhesive.

As shown in the DSC curves in Figure 5a, both soybean meal (SM) and the OTS exhibited characteristic thermal behavior. The SM thermogram displayed a typical exothermic peak of protein thermal denaturation, occurring between 140 °C and 160 °C [34], with its peak temperature at 147 °C. During curing, the OTS adhesive showed a clear, broad, and unimodal exothermic peak, which implies that OTN and SM underwent an integrated curing reaction in this stage. Curing initiates at 116 °C, which is identified as the system’s initial curing temperature. This curing temperature proves to be significantly below the initial transition temperature for the soybean meal protein alone. The maximum exothermic peak was reached at 138 °C, suggesting that a vigorous polycondensation reaction occurs between tannin and soybean meal at this temperature. The cross-linking reaction seemed complete by reaching 150 °C.

TG and DTG analyses were conducted to evaluate the thermal stability of the OTS, and the corresponding curves are shown in Figure 5b,c. The adhesive’s thermal degradation proceeds in three stages. Stage one occurs between 30 °C and 130 °C. Within this range, weight loss began at 80 °C, and the peak mass loss rate is observed at 105 °C. This stage is mainly ascribed to the loss of water and low-molecular-weight volatile compounds present in this system. The second stage is from 130 °C to 200 °C, which is the plateau region of the curve, indicating that the mass of the adhesive remains basically unchanged during the heating process. This may be because the adhesive gradually cures and reaches a thermally stable state at this stage. The third stage is from 200 °C to 650 °C. In this temperature range, obvious thermal decomposition occurs within the cured adhesive matrix, and decomposition onset is about 220 °C. This stage is also the main stage for mass loss, attributable to the destruction of the adhesive network structure by the high temperature. A relatively wide peak appears in the DTG curve, suggesting a relatively slow thermal decomposition rate of the adhesive. From 650 °C to 800 °C, the relatively small mass loss in this region further indicates the good thermal stability of the cured oxidized tannin–soybean meal adhesive.

## 4. Discussion

The combined structural, performance, and thermal results indicate that the improvement of the OTS mainly originated from the formation of a quinone–amine covalent crosslinked network between laccase-oxidized tannin and soybean meal proteins. In this system, oxidized tannin did not merely serve as a blended polyphenolic additive, but functioned as a reactive crosslinking precursor. This distinction is important because the improved wet bonding performance was not simply due to physical filling or hydrogen-bond reinforcement but to the establishment of a more compact and hydrolysis-resistant cured network.

This interpretation is generally consistent with previous studies showing that tannin-derived components can enhance soy-based adhesives by participating in network formation. However, many reported strategies relied on formaldehyde-based reagents, hydroxymethylation treatments, or other chemical modifiers to increase tannin reactivity. In contrast, the present work employed laccase to activate waxberry tannin under mild conditions, thereby avoiding aldehyde-based crosslinkers and additional chemical oxidants. From this perspective, the present system is not only formaldehyde-free, but is also mechanistically characterized by the formation of enzymatically generated quinone structures followed by quinone–amine coupling with soybean meal proteins.

Adhesive performance depended on achieving a proper balance among oxidation degree, formulation ratio, and curing conditions. Moderate oxidation, an appropriate OTN:SM ratio, and sufficient hot-pressing temperature favored effective quinone–amine crosslinking, whereas excessive oxidation, excessive tannin content, or insufficient curing were unfavorable for the development of a stable network and overall water resistance. This interpretation was also supported by the thermal behavior of the OTS, which indicated a coupled curing process and relatively good stability before major thermal degradation.

Although the OTS showed improved bonding performance and water resistance, some limitations remain. Residual free amino groups were still detected, indicating that the reaction between oxidized tannin and soybean meal was not complete. In addition, the preparation conditions were mainly optimized by single-factor experiments, and interactions among different factors were not considered. Future work may focus on multivariable optimization, more in-depth characterization of the cured network, and systematic evaluation of practical properties such as storage stability and wood penetration behavior.

## 5. Conclusions

In this study, a formaldehyde-free oxidized tannin–soybean meal adhesive (OTS) was successfully developed through the laccase-mediated oxidation of waxberry tannin, followed by quinone–amine crosslinking with soybean meal proteins. The main conclusions are as follows:

(1) The optimal preparation conditions were a laccase dosage of 10% based on TN mass, an oxidation time of 6 h at 40 °C, an OTN:SM mass ratio of 0.5:1, and a hot-pressing temperature of 160 °C. Under these conditions, the plywood bonded with OTS exhibited a wet shear strength of 0.85 MPa after 3 h immersion in water at 63 °C, representing a 136% increase over that of the neat soybean meal adhesive, while the boiling-water shear strength reached 0.61 MPa, indicating markedly improved water resistance. Moreover, under harsh hydrothermal conditions, the OTS exhibited bonding performance comparable to, or slightly better than, that of the commercial UF adhesive.
(2) FT-IR and XPS analyses confirmed that laccase oxidation converted phenolic hydroxyl groups in tannin into quinone-containing reactive structures. During curing, these oxidized tannin structures further reacted with amino groups in soybean meal proteins through quinone–amine reactions, forming C–N and C=N bonds and thereby establishing a covalently crosslinked adhesive network, which was responsible for the improved bonding performance and water resistance.
(3) DSC and TG/DTG analyses indicated that the OTS exhibited good curing reactivity and relatively good thermal stability. The cured adhesive remained relatively stable in the range of 130–200 °C, while obvious thermal decomposition began at about 220 °C. These results further support the formation of a denser and more stable cured structure.

Overall, this work demonstrates that the laccase-mediated oxidation of tannin provides a feasible green strategy for developing high-performance soybean meal-based adhesives under formaldehyde-free conditions.

## Figures and Tables

**Table 1:** Preliminary evaluation of the industrial parameters of the OTS at different times after preparation.

Time After Preparation	Viscosity	Coating Behavior
(h)	mPa·s	-
0	303	Easy to apply with uniform coating
1.0	596	Difficult to apply with uneven coating
1.5	-	Caking occurred and the adhesive could not be applied

**Table 2:** Elemental composition of OTN, SM, and OTS from the XPS full spectrum.

Sample	C1s	N1s	O1s
	%	%	%
OTN	61.09	3.02	37.06
SM	69.47	7.68	22.84
OTS	65.48	5.69	28.83

## Data Availability

The original contributions presented in this study are included in the article. Further inquiries can be directed to the corresponding author.

## Abbreviations

The following abbreviations are used in this manuscript:

SM	soybean meal
TN	tannin
OTN	oxidized tannin
OTS	oxidized tannin–soybean meal adhesive
